# Transient infrared laser exposure modulates calcium activity in cortical dendritic spines

**DOI:** 10.1117/1.BIOS.3.3.035001

**Published:** 2026-08-05

**Authors:** Jacob Hardenburger, George Grow, Pratheepa Rasiah, Mona Gerges, Joel Bixler, Chad Oian, Bryan Millis, Christopher Valdez, E. Duco Jansen, Anita Mahadevan-Jansen

**Affiliations:** a Vanderbilt University, Vanderbilt Biophotonics Center, Nashville, Tennessee, United States; b Vanderbilt University, Department of Biomedical Engineering, Nashville, Tennessee, United States; c Air Force Research Laboratory, Bioeffects Division, JBSA Fort Sam Houston, Texas, United States; d University of Texas Health San Antonio, San Antonio, Texas, United States; e Department of Neurological Surgery, Vanderbilt University Medical Center, Nashville, Tennessee, United States

**Keywords:** dendritic spines, laser–tissue interactions, calcium signaling, photothermal, infrared neural stimulation.

## Abstract

**Significance:**

Infrared neural stimulation (INS) is an optical neuromodulation technique that elicits neural activity through photothermal temperature gradients. Photothermal gradients have been shown to alter neuronal excitability, but the effects on the dendritic spine (DS) structure and function have not been characterized.

**Aim:**

We aim to determine whether INS induces calcium signaling in DS and whether this signaling alters filamentous actin (F-actin).

**Approach:**

We used confocal imaging of cortical neurons expressing a genetically encoded calcium indicator (GCaMP)8f-Syn1 and mCardinal-LifeAct during laser exposure. Fluorescence signals were measured within regions of interest covering the DS. The resulting fluorescence measurements were baseline-normalized, and transient spiking activity in DS was quantified.

**Results:**

Transient thermal gradients induce persistent calcium activity in a fraction of DS, independent of pulse duration but dependent on the absolute temperature and number of laser exposures. There is no relationship between the calcium activity and F-actin. Calcium signaling in DS was not mediated by ionotropic glutamate receptors but depended on extracellular entry.

**Conclusions:**

These results indicate that transient thermal gradients elicit calcium activity in DS, but the induced activity does not alter F-actin, suggesting that transient thermal gradients may be a useful tool for studying calcium dynamics in DS without triggering traditional structural plasticity pathways.

Statement of Discovery This work demonstrates that infrared-induced photothermal gradients elicit calcium activity in dendritic spines that is unrelated to established plasticity-related pathways. This finding supports the safety profile of photothermal neurostimulation technologies for clinical applications and provides a novel method for inducing calcium signaling in dendritic spines, which may be useful for neuroscience research.

## 1 Introduction

Infrared neural stimulation (INS) is a label-free photothermal neurostimulation method that offers improved spatial precision compared with electrical stimulation.^1^ Since its initial demonstration in 2005 by Wells et al.,^2^^–^^8^ INS has been used on various targets in both the peripheral and central nervous systems. It is currently being studied for multiple clinical uses, including nerve injury monitoring,^9^ cochlear^10^ and cortical neuroprosthetics,^11^ and deep brain stimulation.^8^ Despite the advances in the applications of INS, its mechanisms are not fully understood.

The biophysical mechanism behind INS stems from the conversion of infrared light into heat through water absorption.^12^ Shapiro et al.^13^^,^^14^ demonstrated that rapid thermal gradients alter the electrical capacitance of lipid bilayers, creating inward depolarizing currents. The applied thermal gradient also produces several other physiological effects, such as activating heat-sensitive ion channels^15^^,^^16^ and intracellular calcium release from the endoplasmic reticulum and mitochondria^17^^–^^19^ and promoting water transport.^20^ A limited number of studies have explored how INS affects synaptic transmission. In 2010, Feng et al.^21^ found that pulsed laser irradiation induced γ-aminobutyric-acid-mediated inhibitory postsynaptic currents (IPSCs) in cortical neurons following pulsed laser irradiation for 5 min. Yet, other studies have shown that both excitatory postsynaptic currents (EPSCs) and IPSCs are altered by laser-induced heating.^22^ Much remains unknown about how rapid thermal gradients influence synaptic activity and related plasticity.

Synapses are the fundamental unit of neuronal communication. Dendritic spines (DSs) are the primary target of the axon terminal for excitatory synaptic neurotransmission.^23^ Dendritic spines are characterized as dendritic protrusions with a narrow neck and bulbous head. Their shape is essential to their function, as the bulbous head is densely organized with postsynaptic receptors, enabling robust interactions with presynaptic neurotransmitters at the axon terminal. The dendritic spine neck is thin, enabling signal compartmentalization from distinct axonal inputs.^24^^–^^26^ DS morphology plays an essential role in learning and memory.^27^ Alterations to the DS structure are present in Alzheimer’s disease,^28^ Parkinson’s disease,^29^ and schizophrenia.^30^ Structural changes arise from calcium activity patterns within the spine, leading to long-term potentiation or long-term depression through an enzymatic signaling cascade that triggers filamentous actin (F-actin) remodeling.^31^^,^^32^ Plasticity-related calcium transients are typically mediated through ionotropic glutamate receptors (iGluRs), namely, N-methyl-D-aspartate receptors (NMDAr) and α-amino-3-hydroxy-5-methyl-4-isoxazolepropionic acid receptors (AMPAr). There are several temperature-dependent mechanisms that could affect DS calcium signaling, including accelerated AMPAr kinetics,^33^ intracellular calcium release,^34^ and transient receptor potential (TRP) expression on DS.^35^ The effect of thermal gradients on synaptic activity has only been studied using electrophysiological techniques, which are not selective for calcium.

In this study, we evaluate whether INS can induce localized calcium signaling in DS and whether such activity influences F-actin dynamics in DS. We used high-resolution confocal microscopy to monitor calcium dynamics and F-actin concentration in the DS of primary cortical neurons following INS. The data presented demonstrate that transient photothermal gradients induce persistent calcium activity in a fraction of DS cells, distinct from somatic activity, and that this activity depends on the magnitude, duration, and number of applied thermal gradients. Despite the ability of INS to elicit calcium transients within DS, we observe no relationship between the activity and the level of F-actin expression. However, the infrared (IR)-evoked calcium response in DS depends on external calcium but is not mediated through iGluRs.

## 2 Methods

### 2.1 Primary Cortical Neuron Culture

The culturing protocol is based on the E18 Primary Kit Neuron Culture Protocol from Transnetyx Tissue.^36^^,^^37^ One day before plating, 14-mm glass-bottom imaging wells in 35-mm Petri dishes (P35G-0-14-C, MatTek, Emu Plains, Australia) were coated with poly-D-lysinehydrobromide (A3890401, Thermo Fisher, Waltham, Massachusetts, United States) at a concentration of 50  μg/mL for 12 to 15 h in a sterile hood. At the conclusion of the coating period, the dishes were washed three times with sterile deionized water and dried for 2 h. Dissected cortical tissue was acquired from Transnetyx Tissue (KTSDECX). The cortices were incubated in a papain dissociation solution (2  mg/mL papain in calcium-free Hibernate E) for 10 min. Following digestion, the cells were dispersed by triturating the solution using a fire-polished Pasteur pipette. The dissociated cells were passed through a 100-μm cell strainer to remove any remaining tissue fragments. The cell suspension was centrifuged at 1100 rpm for 1 min. The supernatant was discarded, and the cells were resuspended in neuronal growth media containing 97.75% Neurobasal Plus (A3582901, Thermo Fisher), 2% B-27 Plus (A3582801, Thermo Fisher), and 0.25% GlutaMAX (35050061, Thermo Fisher). Cells were seeded at a concentration of 100 to 120  cells/μL, resulting in a plating density of ∼325 to $390\;\mathrm{cells}/{\mathrm{mm}}^{2}$. Following plating, the dishes were placed in an incubator at 37°C and 5% ${\mathrm{CO}}_{2}$. An additional 1.5 mL of growth media was added to each dish 1 h after plating. The neurons were fed every 3 days by replacing 1 mL of the medium with fresh growth medium until experimental use, between 14 and 21 days *in vitro*.

### 2.2 Transfections

To monitor DS calcium signaling, neurons were sparsely transfected with GCaMP8f-syn1^38^ (Plasmid #162388, Addgene, Watertown, Massachusetts, United States) using Lipofectamine LTX with PLUS (Thermo Fisher, 15338100) 7 days after plating. Transfection complexes were made by adding 500 ng of GCaMP8f-syn1 plasmid, 0.5  μL PLUS reagent, and 2.5  μL Lipofectamine LTX to 100  μL Neurobasal Plus Media without serum, per 35-mm Petri dish, resulting in a DNA:LTX ratio of 1:5. The mixture was left to incubate for 10 min to facilitate lipid–DNA complex formation. Next, the solution containing the lipid–DNA complexes was diluted by adding 100  μL Neurobasal Plus media, which was then added to the imaging well.

To concurrently monitor calcium dynamics and F-actin polymerization, neurons were co-transfected with 250 ng of GCaMP8f-syn1 plasmid, 250 ng of mCardinal-LifeAct-7^39^ (Plasmid #54663, Addgene) plasmid, 0.5  μL of PLUS reagent, and 2.5  μL of Lipofectamine LTX to 100  μL of Neurobasal Plus Media without serum, per 35-mm Petri dish. The remaining steps follow the protocol described above.

### 2.3 Infrared Laser Exposure

Infrared laser light at 1875 nm was supplied by a custom-built 5-W diode laser (Innovative Photonic Solutions, Plainsboro Township, New Jersey, United States) and coupled into a 400-μm diameter, 0.22 NA multimode optical fiber (Ocean Optics, QP400-1-VIS-NIR, Orlando, Florida, United States). The fiber was positioned colinearly with the imaging optical axis in the culture medium, 200  μm above the imaging plane, using a micromanipulator (Fig. S1 in the Supplementary Material). Two stimulation protocols were tested: a single laser exposure (SE) and a series of five exposures spaced 5 s apart (ME). Within these protocols, two pulse parameters were evaluated: a single 8-ms continuous pulse (SP)^20^ and a train of 250-μs pulses delivered at 200 Hz for 500 ms (PT).^40^^–^^42^ These exposure protocols will be referred to as single pulse single exposure (SPSE), single pulse multiple exposures (SPMEs), pulse train single exposure (PTSE), and pulse train multiple exposures (PTMEs). Neurons were exposed to irradiation under two basal temperatures, either at 30°C or 18°C, to evaluate whether neuronal calcium responses depended on the absolute temperature rise or on the transient stimulus. The radiant exposure required for stimulation was empirically determined by increasing the laser power until ${\mathrm{Ca}}^{2+}$ signaling was observed. Laser pulse parameters, basal temperatures, and nomenclature are summarized in Table 1. Laser pulse power was calibrated by measuring the average power output for each pulse condition and calculating pulse energy using the repetition frequency of the laser. These measurements were conducted at the sample plane using a power meter (EMP 5000, Molectron - Coherent, Saxonburg, Pennsylvania, United States) to replicate the geometry during stimulation. The reported radiant exposure at the fiber was calculated by dividing the laser pulse energy by the fiber diameter.

**Table 1:** Laser exposure protocols.

Protocol nomenclature	Pulse width (ms)	Total pulses	Radiant exposure ($J/{\mathrm{cm}}^{2}/\text{pulse}$)	Basal temperature (°C)
SPSE	8	1	18.5	30
SPME	8	5	18.5	30
PTSE	0.25	100	0.7	30
PTME	0.25	500	0.7	30
${\mathrm{PTSE}}_{\text{Sub}}$	0.25	100	0.7	18
${\mathrm{PTME}}_{\text{Sub}}$	0.25	500	0.7	18
${\mathrm{PTSE}}_{\text{Thresh}}$	0.25	100	0.91	18
${\mathrm{PTME}}_{\text{Thresh}}$	0.25	500	0.91	18

### 2.4 Microscopy

#### 2.4.1 Confocal imaging

**Fig. 1:**
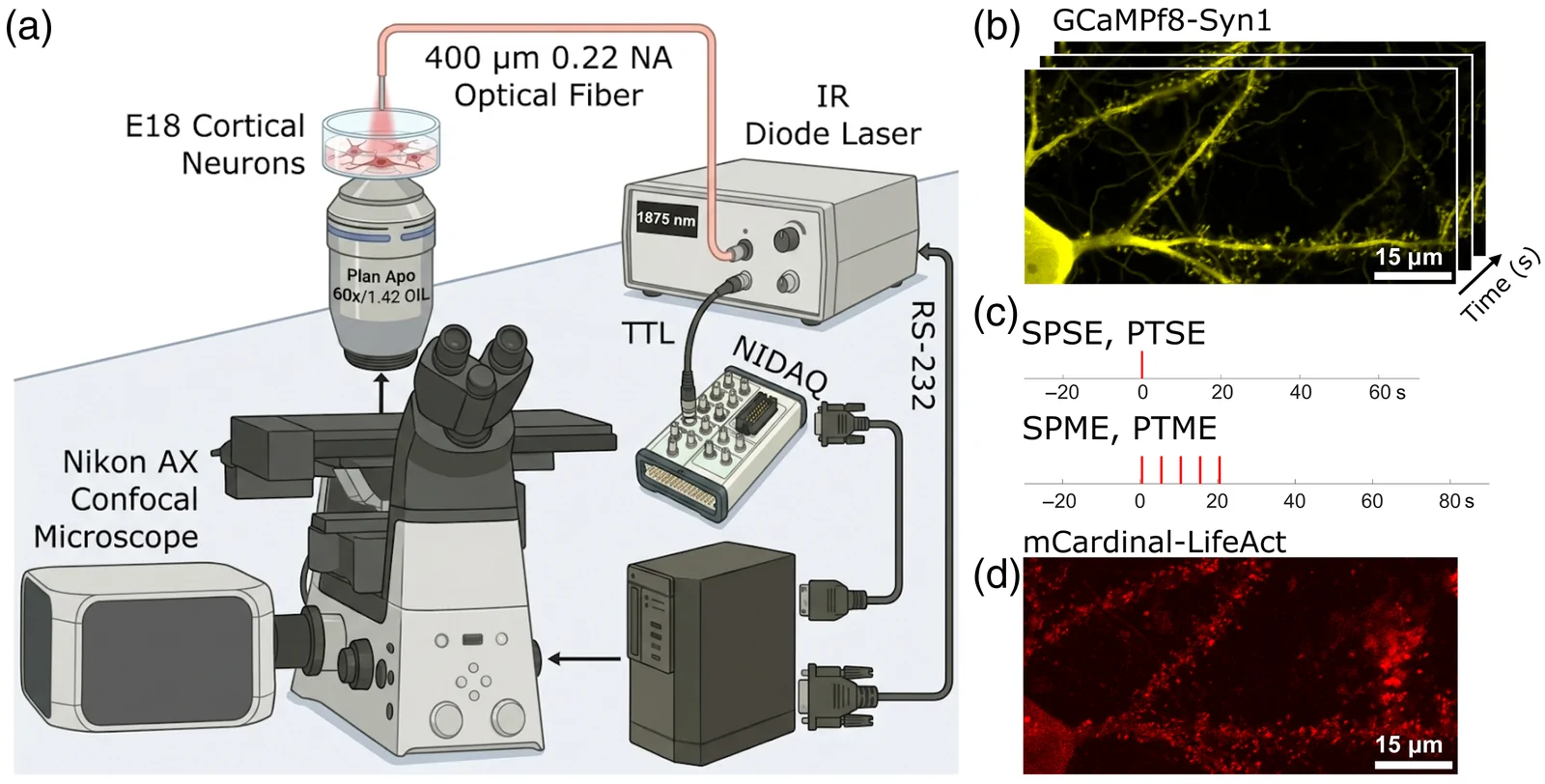
(a) Experimental setup for imaging neuronal responses to infrared laser exposure. The schematic was drafted by the author and visually re-rendered using Google Gemini. (b) Representative time series image stack of a neuron expressing GCaMP8f-syn1. (c) Stimulation protocols employed in the study: the vertical red lines indicate the time at which laser stimulation was applied. (d) Representative image of a neuron expressing mCardinal-LifeAct in DS.

Before imaging, the growth media were aspirated and replaced with electrophysiology solution containing 133 mM NaCl, 4 mM KCl, 1 mM ${\mathrm{MgCl}}_{2}$, 2 mM ${\mathrm{CaCl}}_{2}$, 1.5 mM TES sodium salt, and 11 mM D-glucose. Calcium imaging was conducted using a Nikon-AX inverted confocal microscope [Fig. 1(a)]. A stage incubator (Tokai-Hit) was set to 37°C to maintain the neurons at an elevated temperature; the ambient room temperature was 18°C, resulting in the temperature of the dish media settling near 30°C, as measured with an external thermocouple, consistent with prior *in vitro* INS studies conducted at reduced or even room temperature.^16^^,^^20^^,^^43^^,^^44^ The incubator was turned off for experiments performed at room temperature. A 60×, 1.42 NA oil immersion objective was used to visualize neurons under Nyquist sampling conditions, yielding a pixel size of 82 nm and a resolution of 164 nm. Neurons expressing GCaMP8f-syn1 were excited using 488-nm light, and fluorescence emission was between 500 and 635 nm. Neurons were imaged continuously at 7.5 Hz using a resonant scanner with a four-frame average to monitor neuronal activity before, during, and after INS. Only neurons with spontaneous action potential activity detected using GCaMP8f were selected for experiments. Imaging was performed over 100 or 120 s, depending on the stimulation protocol [Figs. 1(b) and 1(c)], and stimulation was applied 30 s into imaging. Pharmacological blockers (Table S1 in the Supplementary Material) were used to investigate the origin of the calcium response during imaging. All blockers were freshly prepared with dimethyl sulfoxide and used within 1 week of preparation.

Dendritic spine calcium activity and F-actin dynamics were measured over 80 min to monitor actin dynamics associated with structural plasticity that typically occurs over minutes to hours.^45^^,^^46^ First, a 120-s video of the pre-stimulation GCaMP activity was captured, and an image of the mCardinal-LifeAct signal excited at 561 nm and detected between 640 and 750 nm was captured to measure the level of pre-stimulation F-actin expression. Next, the calcium reactions to the PTME protocol were imaged following the abovementioned calcium imaging protocol. A post-stimulation snapshot of the mCardinal-LifeAct signal was acquired at the conclusion of the PTME protocol. Then, every 10 min, a 120-s video of the calcium activity was collected, and an image of the mCardinal-LifeAct signal was captured without applying additional laser exposures. Latrunculin A (LatA) was applied to the cells at 20  μM as a positive control to destabilize actin polymerization and validate that mCardinal-LifeAct was sensitive to actin reorganization and that the resulting reorganization was detectable under confocal microscopy [Fig. 5(f) and Fig. S4 in the Supplementary Material].^47^

#### 2.4.2 Thermal modeling

**Fig. 2:**
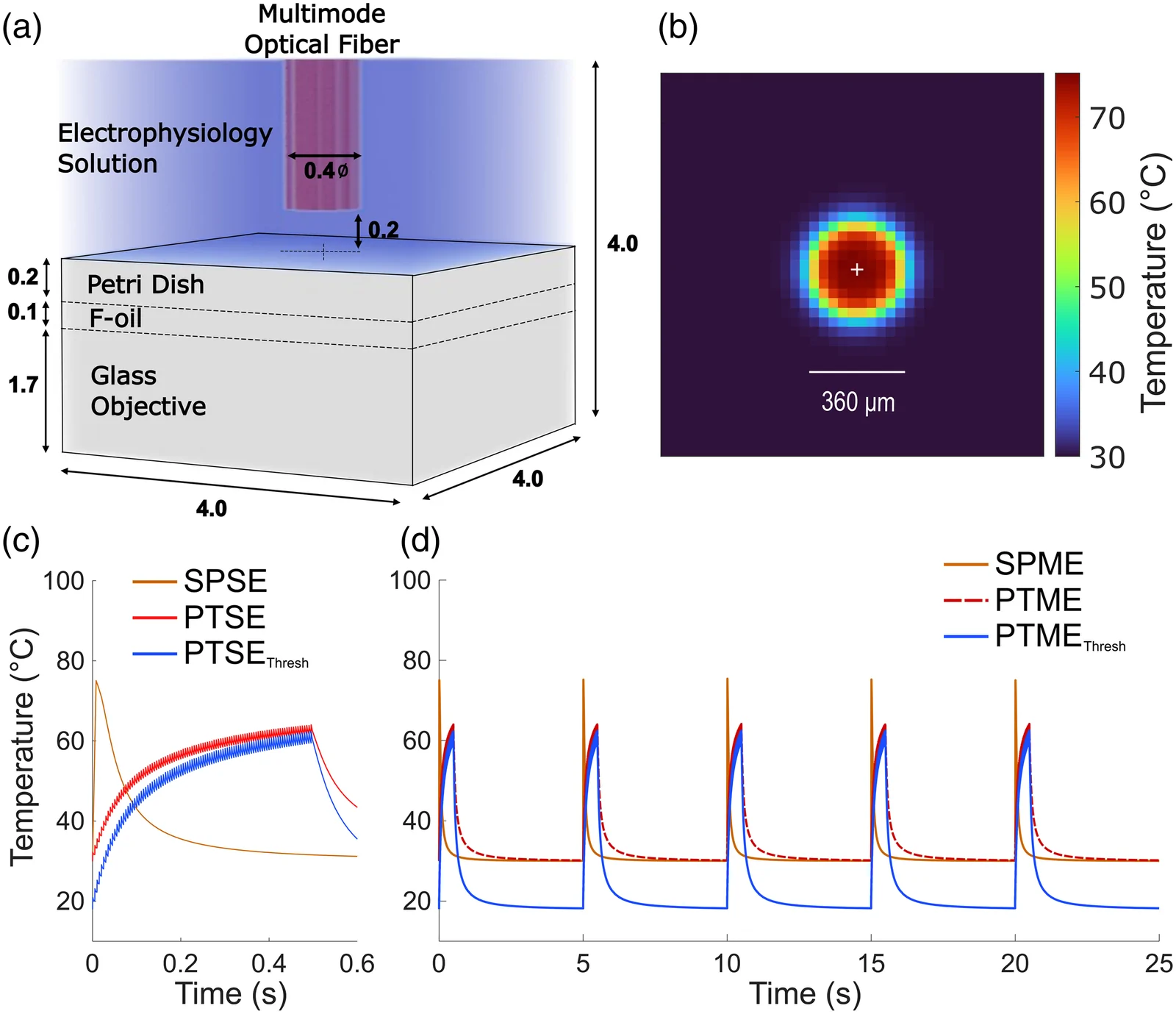
(a) Thermal model geometry (not to scale, dimensions in millimeters). (b) Spatial heating profile at the imaging plane immediately following a single 2.91-W, 8-ms laser pulse. The white cross (+) indicates the location of the imaging field relative to the heating profile. (c) and (d) Temperature profiles for each exposure protocol. The pulse train was simulated at two base temperatures: 18°C (${\mathrm{PTSE}}_{\text{Thresh}}$ and ${\mathrm{PTME}}_{\text{Thresh}}$) and 30°C (PTSE and PTME).

The thermal rise from laser irradiation was modeled using the Scalable Effects Simulation Environment v2.6.0 (SESE) (NanOhmics, Austin, Texas, United States), a program for simulating physical processes involving radiative transfer and thermal diffusion in heterogeneous structures.^48^ The SESE program uses Monte Carlo ray tracing to simulate light propagation throughout a material. Absorbed energy distribution is assumed to be converted entirely into heat and used as input to the Fourier heat conduction equation. A computational mesh consisting of rectangular nodes was generated based on the expected spatial temperature gradient. The mesh was initialized as a 4  mm×4  mm×4  mm block, with voxel sizes of 40  μm in the horizontal direction and 10  μm in the axial direction. The total domain size was chosen because these dimensions allowed for less than a 0.1% change in boundary temperature during the simulation. The light propagation out of a 400-μm optical fiber, 200  μm above and normal to the bottom of the Petri dish, was calculated by the model. The model geometry was assumed to be accurately characterized by five domains [Fig. 2(a)]. Optical and thermal properties are estimated by the bulk material properties shown in Table 2. Dirichlet boundary conditions set at the reference temperature were defined to simulate heat flux across the boundary. The cells were assumed to be located at the center of the irradiated area in the first layer above the Petri dish boundary [Fig. 2(b)].

**Table 2:** Material properties used in the thermal model.

λ=1875 (nm)	Refractive index (n)	Optical absorption $\left(\mu_{a})(m^{-1}\right)$	Density (ρ) ($\mathrm{kg}/m^{3}$)	Specific heat (c) (J/kg K)	Thermal conductivity (k) (W/mK)
Multimode fiber low OH (${\mathrm{SiO}}_{2}$)	1.44	0	2200	703	1.40
Electrophysiology media ($H_{2}O$)	1.32	2540	997	4182	0.70
Petri dish ($B_{2}O_{3}$)	1.50	0	2200	830	1.10
F-oil	1.50	0	1059	1930	0.13
Objective (${\mathrm{SiO}}_{2}$)	1.44	0	2200	703	1.40

### 2.5 Data Analysis

#### 2.5.1 Image processing and signal measurement

Following the acquisition, the time-series data were deconvolved using an automatic deconvolution in NIS-Elements to improve the image stack’s signal-to-noise ratio (SNR). Image segmentation was performed using Imaris 10.2.0. A maximum intensity projection was computed to reduce data dimensionality. Regions of interest (ROIs) covering the soma and DS were created using the machine learning pixel classifier within the Surfaces function in Imaris. The pixel-classifier required semi-manual editing, requiring the user to iteratively mark the signal and background until masks covering the DS and soma were segmented. Following segmentation, the ROI were exported as Tagged Image Format files.

A Custom MATLAB script was used to measure the mean fluorescence intensity under the ROI for each imaging protocol. The raw image stack is loaded into MATLAB and smoothed using a three-dimensional Gaussian filter with a σ(x,y,t)=[2.5,2.5,0.2] kernel to suppress noise by blurring the image while preserving the temporal signal. Then, the mean fluorescence intensity is measured under each frame’s ROI. The distances of each spine from the parent soma were calculated by computing the Euclidean distance between the centroids of the soma and DS.

Multimodal affine registration was used to register the mCardinal images to a maximum intensity projection of the pre-stimulation GCaMP signal in the extended imaging experiments to account for any slight movement that occurred over the duration of the experiment.^49^ The registration transform was acquired using the *imregtform* MATLAB function. The transform was then applied to the binary segmentation containing the ROI covering the DS using the *imwarp* MATLAB function. The mCardinal signal was then measured underneath the ROI.

#### 2.5.2 Signal feature extraction

Several metrics, including mean fluorescence rise, spike height, and spike duration, were obtained to quantify the effect of INS in the somata and DS. All signals were normalized to the minimum intensity observed within 15 s before stimulation to measure fluorescence changes relative to baseline. The change in somatic fluorescence following INS was measured by averaging the normalized fluorescence value over 5 s following the final IR exposure. A median threshold was then used to delineate somatic phenotypes.

Neuronal spiking activity was quantified in MATLAB using a derivative-based peak-finding procedure. The first temporal derivative was computed for each calcium signal, and spiking events were detected as positive outliers within the derivative. The SNR was much lower in DS than in the soma. Therefore, to prevent false negatives in somatic signals while excluding false positives from DS signals, the threshold for outlier detection was set to be one standard deviation of the derivative of the somatic fluorescence signals and three times the standard deviation for DS signals. Local maxima in the underlying fluorescence signal within five samples of an outlier in the derivative were identified as spiking events. Rapid thermal gradients induce changes in the refractive index of the imaging media, creating a thermal lens that defocuses the image, leading to a sharp decrease in fluorescence intensity during stimulation.^50^ The relaxation of the thermal lens and the concurrent return of the fluorescence signal appeared as a false positive in outlier detection. Therefore, peaks were only counted during steady-state thermal distribution, and peaks occurring within 1 s of stimulation were discarded. Spike amplitudes and full-width at half-maximum (FWHM) were quantified by subtracting the baseline fluorescence signal captured using a 5-s moving median filter from the raw fluorescence signal and then extracting the peak height and FWHM from the residual signal. Spiking activity in DS was marked as back-propagating action potentials (bAP) if the spike occurred within 1 s of a somatic spiking event; otherwise, spikes were determined to be independent calcium spikes (ICSs).

mCardinal-LifeAct fluorescence measurements were normalized to the pre-stimulation measurements of the actin signal to analyze the change from baseline. Changes in F-actin expression were analyzed in aggregate and striated, based on activity observed in the GCaMP signal over the duration of the extended imaging experiments. The distribution of spines showing elevated activity is skewed, as most spines show no spiking activity [Fig. S3A in the Supplementary Material). Therefore, spines were analyzed by their level of spiking activity and separated into three groups using two thresholds: no activity (signal with zero ICS) and low activity if they displayed between one and five ICS, and spines above five ICS were labeled as high activity.

## 3 Results

### 3.1 Thermal Rise During Stimulation

The SESE environment was used to simulate the temperature rise during irradiation using the experimental geometry and laser parameters required to induce calcium signaling in dendritic spines. The irradiated spot shows a Gaussian thermal profile with a $e^{-2}$ diameter of 360  μm. The maximum thermal increase occurs at the center of the irradiated spot that covers the imaging field for each pulse parameter [Fig. 2(b)]. A single $18.53\text{-}J/{\mathrm{cm}}^{2}$, 8-ms laser pulse produces a 45°C increase above the baseline temperature of 30°C. For the pulse train protocols, radiant exposures of 70 and $91\;J/{\mathrm{cm}}^{2}$ result in maximum increases of 34°C and 42°C for the PTSE and ${\mathrm{PTSE}}_{\text{Thresh}}$, respectively, [Fig. 2(c)]. Individual pulses within the pulse train generated thermal transients of 2.2°C for PTSE and 2.8°C for ${\mathrm{PTSE}}_{\text{Thresh}}$, with each successive laser pulse compounding over the duration of irradiation. The thermal gradient under the multiple exposure regime fully relaxed to baseline following each laser exposure [Fig. 2(d)]. There was no difference in peak temperature between single and multiple laser exposures for both single pulses and pulse trains. These simulations establish the thermal dynamics that drive the observed calcium signal in the remainder of the study.

### 3.2 Anatomical Variation in Calcium Response

**Fig. 3:**
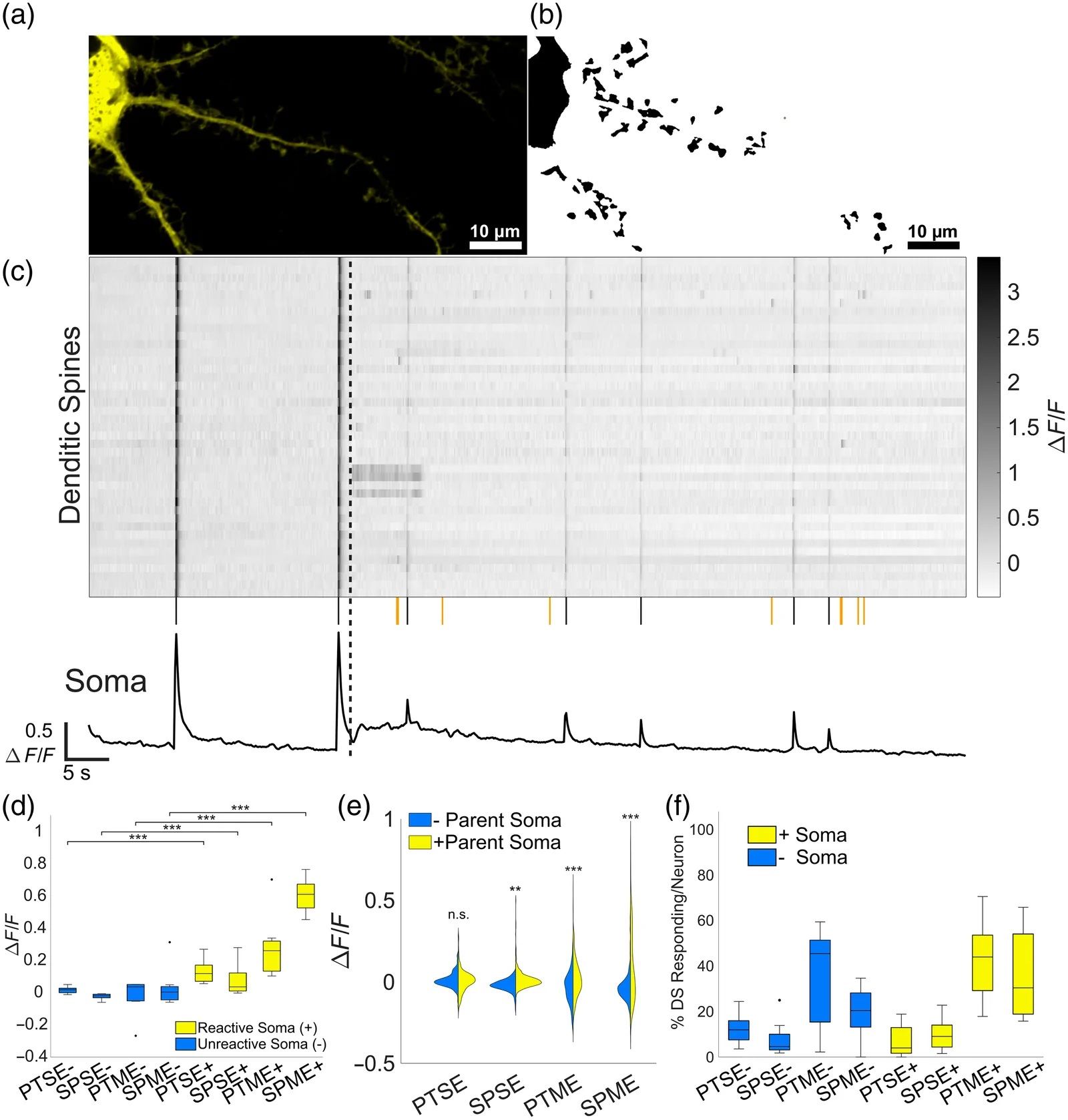
DS reactions differ from soma in response to INS. (a) Image of a neuron expressing GCaMP8f-syn1. (b) Regions of interest used to measure GCaMP8f-syn1 signal in soma and DS in panel (a). (c) Representative GCaMP fluorescence data recorded from the neuron in panel (a) using ROI in panel (b) during the SPSE protocol. Individual DS fluorescence data are shown with the heatmap, and the corresponding parent soma response is plotted below. In the heatmap, each row represents an individual DS located on the dendrites. The dashed line indicates the time at which the stimulus was delivered. Tick marks above the somatic fluorescence trace indicate peaks in activity: black for bAPs and red for ICS in DS. (d) Average fluorescence rise in soma 5 s following stimulation and separated by response phenotype, Wilcoxon rank sum test among phenotypes in the same exposure protocol. Fifteen neurons were tested per exposure protocol, across four biological replicates from two separate cell harvests (N=8 and 7 unreactive (−) and reactive (+) neurons, respectively). (e) Average fluorescence rise in DS 5 s following stimulation, grouped by somatic phenotype. One-way ANOVA with Bonferroni correction and Cohen’s d=0.08±0.15, 0.6±0.15, 0.49±0.15, and 0.74±0.15 for the PTSE, SPSE, PTME, and SPME protocols, respectively (reactive soma: n=332, 393, 291, and 262 DS and unreactive soma: n=382, 358, 423, and 489 DS for the PTSE, SPSE, PTME, and SPME protocols, respectively). (f) Fraction of DS responding per neuron grouped by somatic phenotype and exposure protocol, Wilcoxon rank sum test among response phenotypes in the same exposure protocol. **p<0.01 and ***p<0.005.

Infrared-mediated calcium responses in DS differ from those in the soma [Fig. 3(a)]. Somatic calcium signals display two phenotypes following stimulation, while DS exhibit elevated transient calcium activity independent of somatic phenotype. Reactive cells exhibit a basal increase in somatic fluorescence following stimulation, while unreactive cells show no change from baseline [Fig. 3(b)]. The somata of neurons in the unreactive group show a fluorescence increase of 0.2±1.45% (mean ± SEM) following stimulation across all protocols. Reactive neurons show a 13.3±2.9% and 7.8±3.9% increase in fluorescence for the PTSE and SPSE stimulation protocols, respectively. Despite the nominal difference, there is no statistically significant difference among the responses of neurons in these groups [p=0.28, Wilcoxon rank sum test, Fig. 3(b)]. Applying multiple laser exposures amplified the somatic median fluorescence increase to 28.0±7.8% and 60.7±4.0% for the PTME and SPME stimulation protocols, respectively. The SPME protocol elicited a statistically larger somatic calcium response than the PTME protocol [p=0.028, Wilcoxon rank sum test, Fig. 3(b)]. The DS of neurons exhibiting a somatic calcium response shows a slight increase in fluorescence compared with the DS of unresponsive neurons for each exposure protocol [Fig. 3(c)]. Following laser exposure, DS of unreactive neurons shows a 1.72±0.22% decrease in fluorescence. The DS on reactive neurons show a fluorescence increase of 0.56±0.36%, 1.27±0.27%, 2.11±0.63%, and 8.7±1.3% for the PTSE, SPSE, PTME, and SPME protocols, respectively [Fig. 3(c)]. Despite the marginal increase in DS fluorescence, there is no statistically significant difference between the fraction of DS exhibiting transient activity regardless of somatic phenotype [Fig. 3(d)].

### 3.3 Characterization of IR-Induced Calcium Activity

**Fig. 4:**
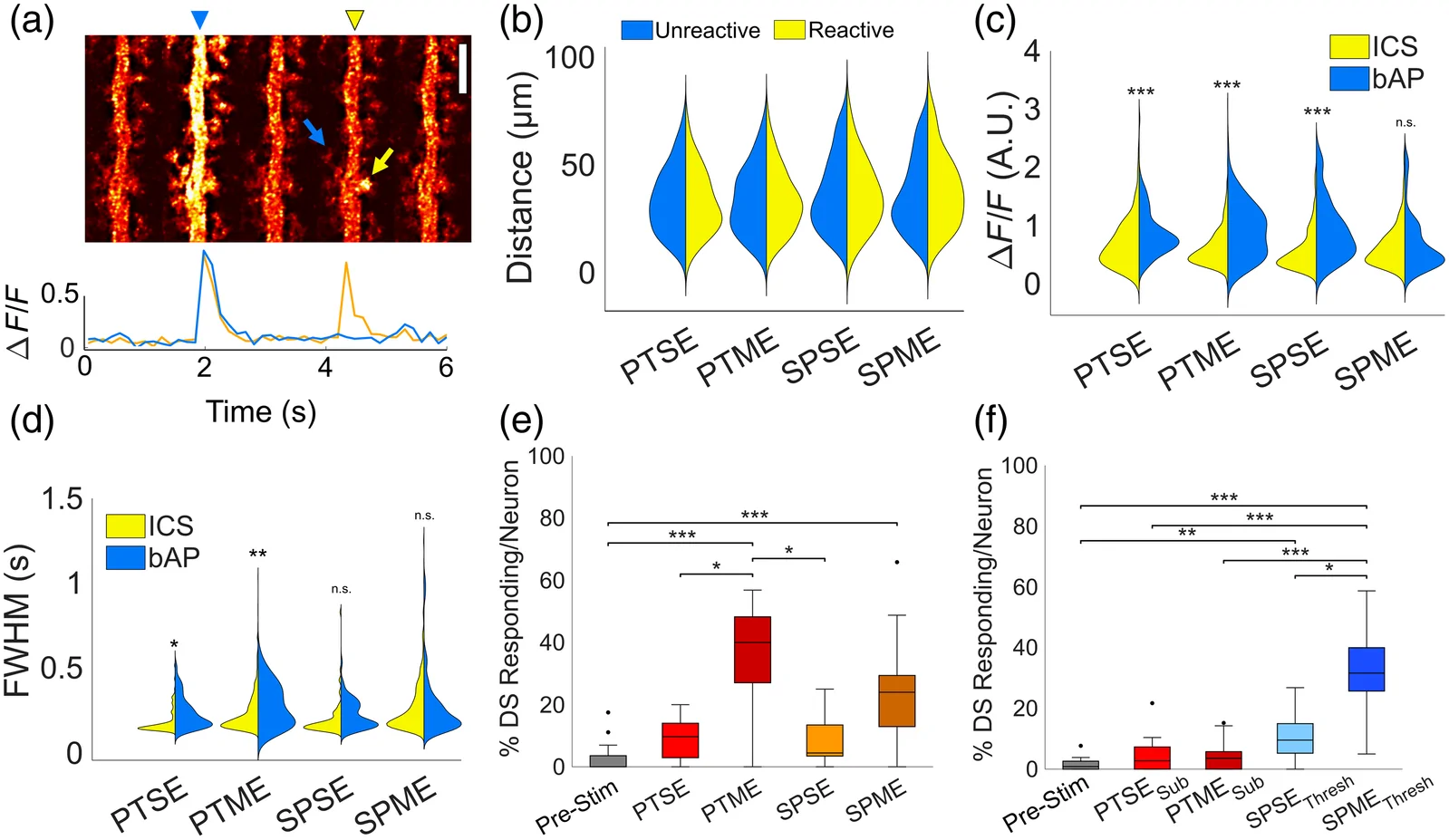
(a) Image series of a dendritic branch showing reactive and unreactive DS following stimulation (scalebar=5  μm). Representative calcium traces of a reactive DS (yellow arrow) and an unreactive DS (blue arrow) showing independent calcium activity. bAP activity (blue triangle) can be seen in both DS signals, while only one spine exhibits increased activity (yellow triangle). (b) Distance of each spine from the soma, separated by reactivity. No intragroup significance one-way ANOVA. (c) Maximum change in fluorescence of DS that exhibits ICS following irradiation, separated by activity phenotype (ICS: n=138, 755, 77, and 456 and bAP: n=129, 118, 59, and 62 for the PTSE, PTME, SPSE, and SPME protocols, respectively). Intragroup significance was tested using a one-way ANOVA with a Bonferroni correction. (d) FWHM of DS calcium spikes separated by activity phenotype. Intragroup significance was tested using a one-way ANOVA with a Bonferroni correction. (e) Fraction of DS responding on each neuron (N=15) following each exposure protocol. Kruskal–Wallis test with Bonferroni correction. (f) Fraction of DS responding on each neuron (N=18) after lowering the dish temperature to 18°C and varying exposure energy. Kruskal–Wallis test with Bonferroni correction. *p<0.05, **p<0.01, and ***p<0.005.

Following INS, transient DS activity occurs stochastically across the dendritic arbor [Fig. 4(a), Video S1, MP4, 5.33 MB [URL: https://doi.org/10.1117/1.BIOS.3.3.035001.s1] and Video S2, Mp4, 9.05 MB [URL: https://doi.org/10.1117/1.BIOS.3.3.035001.s2] in the Supplementary Material). Neuronal maturity showed a weak negative correlation with DS responses to irradiation (Spearman’s ρ=−0.312, p=0.0063, Fig. S2 in the Supplementary Material). However, data were pooled for subsequent analysis as this was not a strong predictor for neuronal reactions. No correlation between the location of reactive spines and their distance from the soma was observed [Fig. 4(b); Pearson’s r ranging from −0.013 to 0.05]. The amplitude of transient ICS following INS is distinctly lower than bAP detected in reactive spines, displaying a 53.9±25.4% fluorescence increase compared with 77.9±35.3% (median ± MAD) increase in bAP amplitude across all exposure types [Fig. 4(c)]. Similarly, transient DS spikes following INS are shorter than bAP-related activity, lasting 189.4±89.1  ms compared with 216.5±89.6  ms, respectively, across all exposure protocols [Fig. 4(d)]. Single laser exposure induces a slight increase in the fraction of DS responding from 2.1±0.73% before exposure to 8.76±1.7% and 8.27±1.9% (mean ± SEM) following the PTSE and SPSE protocols, respectively. However, applying multiple exposures significantly increased the fraction of spines responding following INS to 36.7±4.4% and 24.5±4.2%, respectively [Fig. 4(e)]. Holding laser power constant and reducing media temperature from 30°C to 18°C eliminated DS responses using the ${\mathrm{PTME}}_{\text{Sub}}$ and ${\mathrm{PTME}}_{\text{Sub}}$ stimulation protocols [Fig. 4(f)]. Increasing laser power at lower temperatures restores activity to levels similar to those observed in experiments conducted at 30°C (p=1 between PTME and ${\mathrm{PTME}}_{\text{Thresh}}$, and PTSE and ${\mathrm{PTSE}}_{\text{Thresh}}$). Under the ${\mathrm{PTME}}_{\text{Thresh}}$ and ${\mathrm{PTSE}}_{\text{Thresh}}$ protocols, the radiant exposure required for stimulation was greater than that of the PTME and PTSE protocols (Table 1); however, the absolute temperature increase was similar [Figs. 2(c) and 2(d)].

### 3.4 Concurrent Calcium and F-Actin Dynamics

**Fig. 5:**
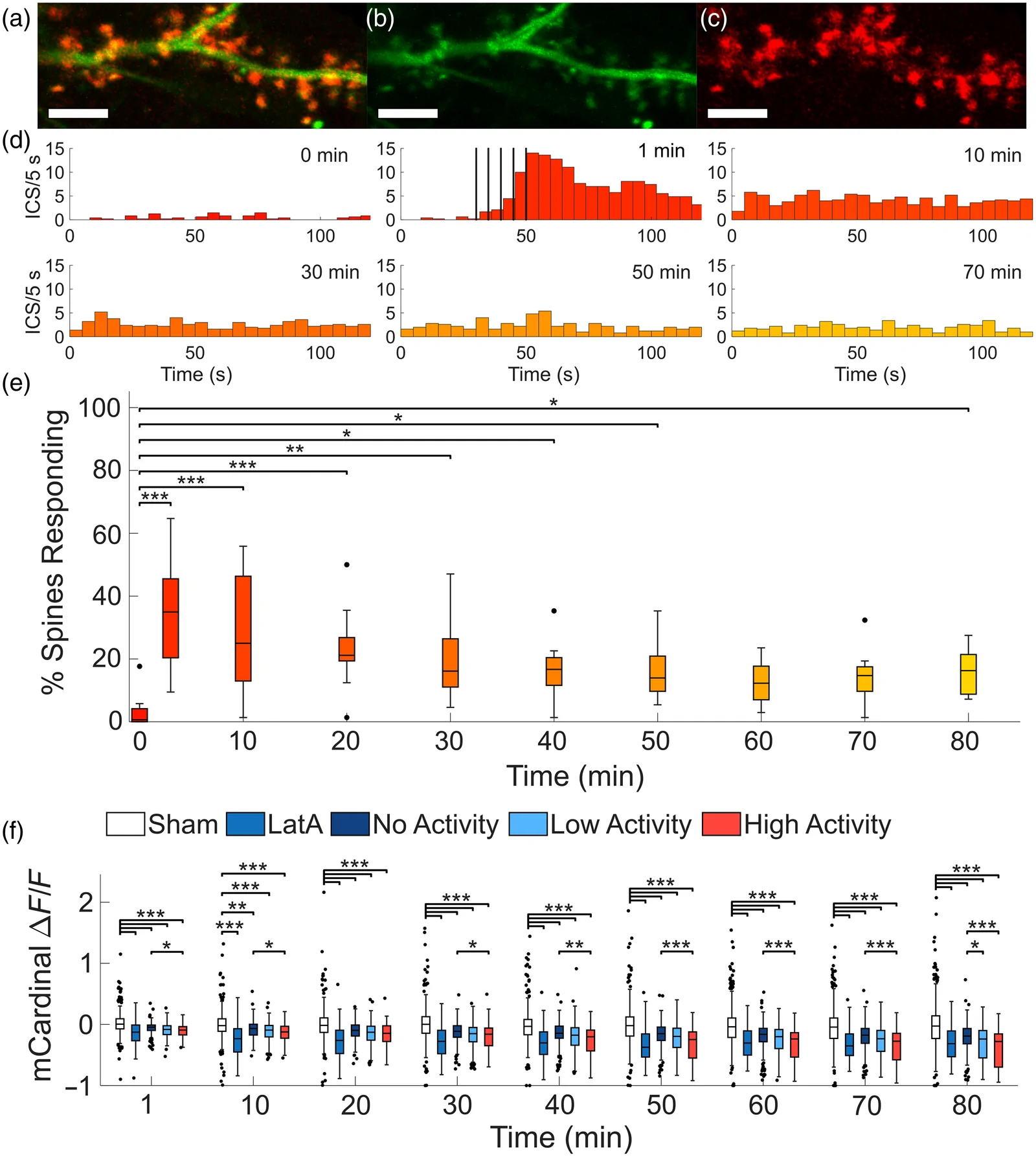
Dendritic spine responses to INS show persistent activity following PTME but do not lead to significant changes in actin expression. (a)–(c) Representative images of dendritic branch co-expressing GCaMP8f-syn1 (b) expressed homogenously across structures and mCardinal-lifeact (c) localized predominantly in DS (scalebar=5  μm). (d) Histograms showing the number of ICS detected during each imaging segment. Vertical lines at 1 min indicate each laser exposure in the PTME protocol. (e) Fraction of DS exhibiting ICS activity for each neuron (N=11) shown at each time point. Kruskal–Wallis test with a Dunnett correction, using activity at 0 min as the control group; *p<0.05, **p<0.01, and ***p<0.005. (f) Change in mCardinal expression from baseline in DS separated by ICS activity (N=8 neurons and n=188, 256, and 118 DS in the non-reactive, low activity, and high activity groups, respectively; sham group: N=6 neurons and n=382 DS; LatA group: N=6 neurons and n=280 DS). Outliers are plotted as dots. Kruskal–Wallis test with a Bonferroni correction; *p<0.05, **p<0.01, and ***p<0.005.

Having shown that thermal gradients can modulate calcium activity to determine whether this activity produces a change in F-actin, we imaged neurons co-expressing GCaMP8f-syn1 and mCardinal-LifeAct in the head and neck of DS [Figs. 5(a)–5(c)]. Following the PTME protocol, spiking activity in DS is greatest following laser irradiation and gradually decreases over the imaging period [Fig. 5(d)]. Dendritic spine spiking activity remains elevated compared with the pre-stimulation observation period over the duration of the imaging period [Fig. 5(e)]. Most spines do not respond, resulting in a skewed distribution with a heavy right tail in the number of ICS observed over the imaging period (Fig. S3A in the Supplementary Material). No correlation between the number of ICS observed and mCardinal fluorescence at each time point (Spearman’s ρ=−0.17±0.03, p<0.0001) was observed. Application of LatA reduced mCardinal intensity by 28±1.4% after 30 min of imaging [Fig. 5(f) and Fig. S4 in the Supplementary Material). Analyzing aggregate DS F-actin expression shows a decrease over the course of the experiment; however, none of the subsequent time points is statistically significant (p>0.26, Kruskal–Wallis with Bonferroni correction). All spines, regardless of activity level, decreased in intensity relative to a sham group of neurons that were not irradiated. Unreactive DS that exhibit no ICS show a decrease in mCardinal fluorescence from 5.9±0.71%, 1 min after stimulation, to a total decrease of 21.2±1.7%, 80 min after stimulation. The most active spines show the largest decrease in fluorescence from 10.8±0.10%, 1 min following imaging to a total decrease of 39.1±2.8% after 80 min [Fig. 5(f)]. The mCardinal fluorescence change in each individual neuron reveals a clear sample dependence [Figs. S3C–S3E in the Supplementary Material). Performing a Kruskal–Wallis test among DS reaction phenotypes for each neuron displays no statistically significant difference in mCardinal expression after applying a Bonferroni alpha correction (p>0.017, Fig. S3F in the Supplementary Material).

### 3.5 Infrared-Induced Calcium Activity Mediated Through Extracellular Entry

**Fig. 6:**
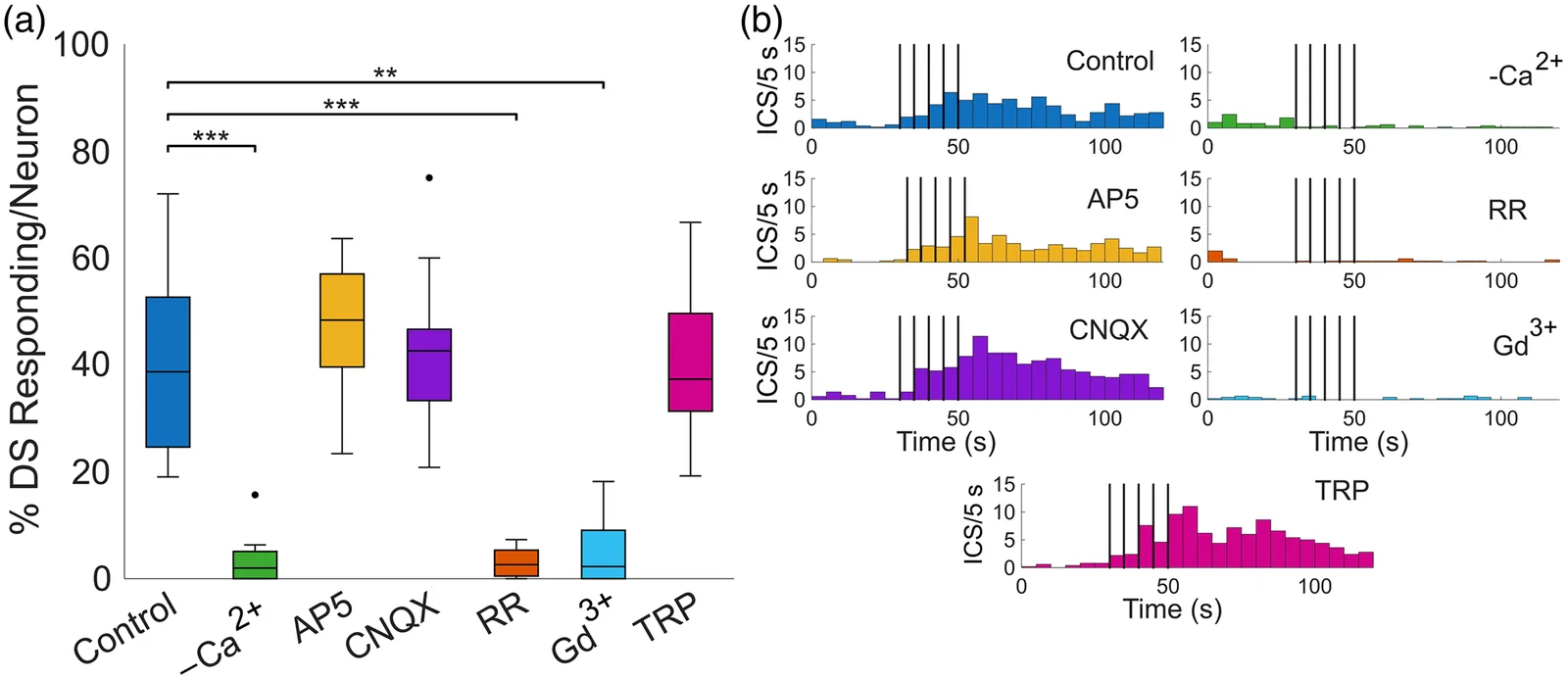
(a) Fraction of dendritic spines responding following the PTME protocol for each tested pharmacologic condition (N=11, 9, 9, 10, 11, 7, and 9 neurons for the control, $-{\mathrm{Ca}}^{2+}$, AP5, CNQX, RR, ${\mathrm{Gd}}^{3+}$, and TRP groups, respectively). Kruskal–Wallis test with Bonferroni correction; *p<0.05, **p<0.01, and ***p<0.005. (b) Histograms showing the number of ICS observed per 5 s during the PTME protocol. Vertical lines indicate the time laser irradiation was applied.

Pharmacologic inhibitors were applied to determine the mechanism behind the calcium entry into the DS (Fig. 6). Removing extracellular calcium from the imaging media abolished spiking activity in the DS, reducing the fraction of spine events from 40.9±5.1% to 3.61±1.7% (mean ± SEM) following a PTME protocol (Kruskal–Wallis test p=0.0029 control to $-{\mathrm{Ca}}^{2+}$). We tested whether ionotropic glutamate receptors were responsible for calcium influx into the cell by applying CNQX to block AMPAr and AP5 to block NMDAr;^21^ both elicited responses similar to the control group (Kruskal–Wallis test: p=0.995 for AP5 versus control; p=1 for CNQX versus control). Application of ruthenium red (RR) significantly reduced spiking activity to 3.28±0.83%, similar to the calcium-free response (Kruskal–Wallis test p=1, RR to CaFree; p=.0027, RR to control). However, RR is known to block various calcium-mediated channels, including TRPV1-4,^51^ TRPA1,^20^ Piezo1,^52^ and calcium homeostasis modulating (CALHM) channels.^53^ Blocking specific TRP channel homologs with individual blockers (Table S1 in the Supplementary Material) failed to stop the calcium response (data not shown).^54^ Combining the TRP blockers also failed to replicate RR’s effect (Kruskal–Wallis test: p=0.007, TRP to RR; p=1, TRP to control). We used gadolinium (${\mathrm{Gd}}^{3+}$) to assess whether mechanosensitive channels such as Piezo1 played a role; it did not block signaling at low concentrations (10, 30  μM; data not shown). Increasing the ${\mathrm{Gd}}^{3+}$ to 100  μM reduced the DS calcium response to 5.4±2.6%, similar to the $-{\mathrm{Ca}}^{2+}$ condition (Kruskal–Wallis test p=1, ${\mathrm{Gd}}^{3+}$ to $-{\mathrm{Ca}}^{2+}$; p=0.021, ${\mathrm{Gd}}^{3+}$ to control).

## 4 Discussion

In this study, we demonstrate that infrared laser-induced transient thermal gradients elicit calcium activity in DS that is distinct from somatic calcium activity (Fig. 3). Our data indicate that the calcium response in DS depends on the magnitude, duration, and number of thermal gradients (Fig. 4). The simulation results indicate that an absolute temperature around 60°C must be met to initiate DS calcium signaling, which persists up to 80 min following stimulation. The pathway responsible for the observed calcium signal does not produce significant changes in mCardinal fluorescence within the limits of our imaging resolution, indicating that photothermal gradients have minimal effects on actin dynamics in DS. The calcium response indicates dependence on extracellular entry rather than intracellular release but it is not mediated by iGluR or TRP channels (Fig. 6).

Although the number of studies is limited, a few groups have demonstrated that laser-induced heating modulates postsynaptic neurotransmission. Prior work from our group found that pulsed laser irradiation, applied at 30 Hz over 5 min, increased the number of inhibitory synaptic events during stimulation of cortical neurons.^21^ Similarly, Entwisle et al.^22^ note a statistically significant increase in synaptic events in brain slices during IR laser irradiation over 1 s. The transient calcium signaling we observe is similar to that reported in these studies, increasing after laser exposure and predominantly asynchronous with the timing of the laser pulse and somatic activity. However, it is difficult to compare our results directly with these studies, as the pulse durations used are at least two orders of magnitude longer than the exposure times used in this study, and the mechanism may depend on pulse duration.^55^ Tolstykh et al.^44^ used a 3-ms, 4.5-mJ laser pulse, more akin to the parameters in this study, and observed that laser-induced heating reduces the number of synaptic events following laser exposure but increases the strength of the synaptic events. The binding sensitivity of the GCaMP8f-syn1 indicator is around 1  μM.^38^ Synaptic events below this threshold would go undetected. The increased strength of synaptic currents following fast thermal gradient (FTG) exposure could lead to more detectable calcium events.^44^

Structural remodeling of dendritic spines is driven by calcium influx, which activates phosphorylation cascades that involve enzymes regulating actin cytoskeletal reorganization.^56^ Provided that the thermal gradients elicited measurable calcium transients in DS, we hypothesized that these events could cause meaningful changes in F-actin expression. Long-term potentiation (LTP) of dendritic spine structural plasticity results in enlargement of the spine head, driven by increased F-actin polymerization.^46^ Typically, LTP involves the coincident binding of glutamate to NMDArs and cell depolarization.^57^ Although we cannot rule out ultrastructural changes in actin remodeling that could occur below the resolution of our imaging system, the absence of a significant increase in mCardinal fluorescence following laser irradiation, together with results indicating that IR-induced calcium entry was not mediated by NMDArs (Fig. 6), indicates that transient thermal gradients do not lead to LTP-related actin reorganization. The decrease in mCardinal fluorescence [Fig. 5(f)] would more likely indicate long-term depression, which results in the shrinkage or removal of DS associated with actin depolymerization.^31^ However, this reduction was not significantly different across DS with varying activity levels, suggesting a global effect across the dendritic arbor unrelated to INS exposure. Thermal effects on the cell membrane may account for both the observed calcium and mCardinal signals. Altered membrane fluidity may induce DS swelling, resulting in less F-actin per unit volume, as reflected by a decrease in mCardinal, and increase membrane permeability to extracellular calcium, thereby increasing calcium influx. Transient IR-mediated heating has been demonstrated to affect the membrane by inducing swelling in both astrocytes^20^ and NG108 cells.^43^

Our initial hypothesis was formulated on the premise that IR heating triggers or potentiates EPSCs.^58^ However, blocking AMPAr and NMDAr, which typically mediate glutamate-related EPSCs, failed to reduce the observed calcium signal in DS, prompting exploration of alternative entry pathways (Fig. 6). Although INS has been shown to affect intracellular calcium release, our results indicate that the calcium signal depends on extracellular calcium entry. Beier et al.^43^ hypothesized that nanopores on the order of 1 to 2 nm may play a role in eliciting cellular responses to INS. However, small nanopores in that range would close within ∼2  ms due to the lipophilicity of the cell membrane.^59^ The persistence of the calcium signal indicates that nanopores are not the primary driver of the calcium signal in this study. Thermally sensitive ion channels, such as TRPV1, TRPV3, TRPV4, and TRPA1, have been shown to contribute to INS responses in neurons and astrocytes.^15^^,^^16^^,^^20^ However, our results indicate that these channels are not involved in the response observed in this study. The results indicated that calcium-permeable channel-mediated mechanisms previously characterized in INS studies are not involved in IR-induced DS signaling. Therefore, we hypothesize that the response is due to either entry through a calcium-selective channel that is sensitive to RR and ${\mathrm{Gd}}^{3+}$ or that the signaling is due to changes in membrane fluidity, initiating a signaling cascade through secondary messengers such as inositol-1,4,5-trisphosphate (${\mathrm{IP}}_{3}$).

One potential channel that could be implicated is the CALHM family of large pore-forming ion channels, which are expressed in cortical neurons and sensitive to extracellular calcium and membrane voltage, as well as to RR and gadolinium (${\mathrm{Gd}}^{3+}$), consistent with our pharmacological observations.^60^ To our knowledge, no reports suggest that CALHM channels are sensitive to thermal gradients. Given the prolonged calcium activity observed in dendritic spines, it is unlikely that heat alone activates these channels; rather, the thermal stimulus may initiate a secondary biochemical cascade that sustains calcium signaling.

At the fundamental level, temperature impacts membrane fluidity,^61^ which could affect DS activity. Our group has demonstrated that INS drives water flux in astrocytes through aquaporins and osmotic pressure; the latter is hypothesized to be related to alterations to the membrane.^20^ Altering water flow at the synapse may affect DS. Although our results indicate that the calcium signal depends on extracellular calcium entry, thermal effects on the membrane could implicate release from intracellular sources through calcium-induced calcium release. Several studies in astrocytes,^20^ HT22 hippocampal neurons,^62^ and NG108 cells^17^ have implicated ${\mathrm{IP}}_{3}$ in intracellular calcium responses to INS. Moreau et al.^62^ suggest that INS triggers ${\mathrm{PIP}}_{2}$ hydrolysis by activating phospholipase C, elevating ${\mathrm{IP}}_{3}$ levels in the cytoplasm, which could initiate calcium release via ${\mathrm{IP}}_{3}$ receptors located on the endoplasmic reticulum or, potentially, the spine apparatus in this study. Depletion of intracellular stores could, in turn, drive extracellular influx via store-operated calcium entry.^63^ Although we did not detect intracellular calcium release under the $-{\mathrm{Ca}}^{2+}$ condition, it could have occurred at sub-detectable levels. The reduction in activity following RR application would support this hypothesis, as RR is also an inhibitor of ${\mathrm{IP}}_{3}R$, suggesting a role for intracellular stores in calcium release.

Fu et al.^64^ demonstrated that INS has differing effects on excitatory and inhibitory cortical neurons, causing positive calcium inflections in excitatory neurons and no effect or decreased effects in inhibitory neurons. Our culture contained both excitatory and inhibitory neuronal subtypes, which could explain the observed differences in somatic responses. Despite the difference in somatic phenotype, DS responses to laser exposure were consistent across both populations. This finding supports our hypothesis that the thermal stimulus induces a universal effect on the membrane rather than relying on transmembrane ion channels. Further, although neuronal maturity was a weak predictor of DS responses, a weak inverse correlation was present for the SPME protocol (Fig. S2 in the Supplementary Material). It is conceivable that older neurons express more transmembrane proteins, making them less susceptible to thermal alterations in the lipid membrane.

The thermal rise reported in prior studies is ∼8°C from room temperature,^22^^,^^44^ much lower than the thermal rise induced in this study. The laser pulse energies applied in this study (Table 1) were selected based on their ability to elicit calcium transients within DS. This would indicate that the signaling observed in previous studies is predominantly attributable to sodium ions or calcium signaling below our detection threshold. Experiments were performed at sub-physiological temperatures (18°C and 30°C), which may have influenced the neuronal responses. However, the surface temperature of the exposed cortex during craniotomy is closer to 30°C,^65^ and therefore, the elevated temperature used in this study is relevant to INS applications for surgical guidance. Our thermal modeling results indicate that the applied laser doses yield peak temperatures of 75°C and 64°C for single-pulse and pulse-train exposure protocols, respectively (Fig. 2). These are larger than the thermal damage threshold for neurons, which is reported to be near 60°C, measured using patch clamp techniques.^66^ It has been noted that photothermal gradients can compromise patch clamp seal resistance and, therefore, may underestimate the true damage threshold.^13^ The cells in this study continued to exhibit spontaneous AP activity after laser irradiation, indicating that they retained functional integrity, although sublethal damage cannot be ruled out. The elevated temperatures used in this study support the hypothesis that the observed calcium signaling is attributable to laser-induced membrane effects.

## 5 Conclusion

To our knowledge, this is the first report to quantify the effects of transient photothermal gradients on dendritic spine calcium activity. Our results demonstrate that transient infrared-induced thermal gradients can evoke calcium signaling within dendritic spines, without eliciting measurable alterations in F-actin expression. The laser parameters employed in this study produced thermal gradients exceeding those typically considered suitable for *in vivo* applications, and the absence of detectable calcium activity at levels below these suggests that actin remodeling is unlikely under clinically relevant conditions. These findings support the safety profile of prospective INS-based neuroprosthetic approaches; however, additional *in vivo* investigations are necessary to confirm these observations. The observed calcium influx exhibits a dependence on extracellular calcium yet is not mediated by canonical ionotropic glutamate receptors or previously characterized transmembrane ion channels implicated in INS responses. Current evidence indicates potential involvement of uncharacterized transmembrane proteins such as CALHM channels or secondary effects arising from thermally induced changes in membrane fluidity. This mechanism introduces the possibility of leveraging INS to modulate calcium dynamics within dendritic spines without activating conventional plasticity-associated pathways. Furthermore, given the inherent biophysical effects of INS on membrane properties, this technique may serve as a label-free investigative tool for probing the relationship between membrane mechanics and dendritic spine function. Identifying the molecular pathway underlying this response will clarify the uses of INS in both fundamental neuroscience research and future clinical applications.

## Supplementary Material

10.1117/1.BIOS.3.3.035001.s01

10.1117/1.BIOS.3.3.035001.s1

10.1117/1.BIOS.3.3.035001.s2

## Data Availability

The measured data supporting the findings of this article are publicly available at 10.6084/m9.figshare.31286167. The raw image files can be made available upon request. The code used for processing is available at https://github.com/PanPandaPan615/Photothermal-Spine-Activity.git.
