# Supplementary material for: Transient infrared laser exposure modulates calcium activity in cortical dendritic spines
Source: Biophotonics Discov. 2026 Aug 5;3(3):035001. doi: 10.1117/1.BIOS.3.3.035001 (PMC13557455; doi:10.1117/1.BIOS.3.3.035001)
Supplement: Supplementary file 1 [file BIOS_003_035001_SD001.pdf]

Supplementary Information

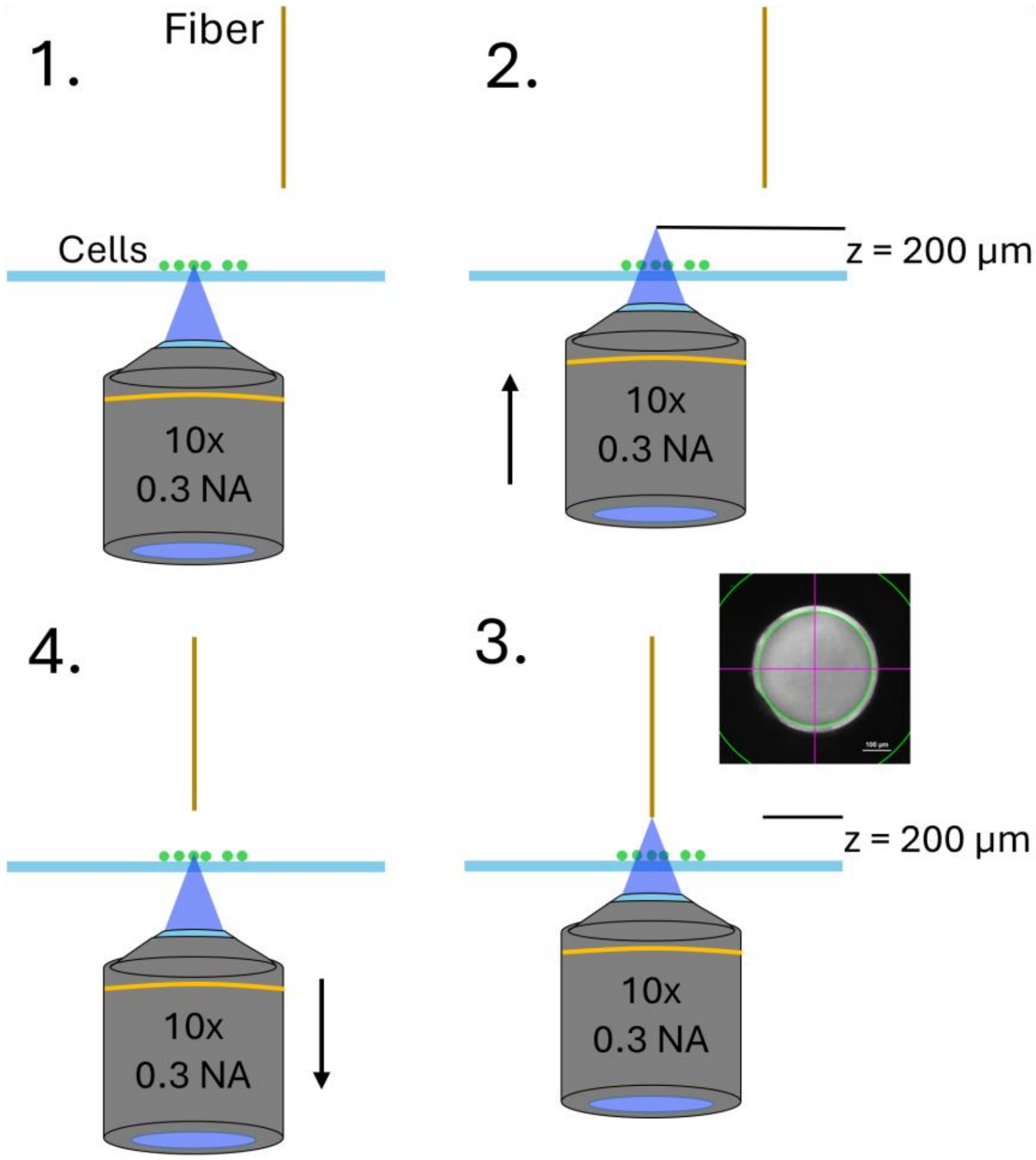

Figure S1: Protocol for placing fiber in the field: 1. Bring cells into focus. 2. Translate focal plane up 200 μm. 3. Position coaxially with the imaging objective shown by inset image. 4. Move focal plane down 200 μm bringing the cells back into focus.

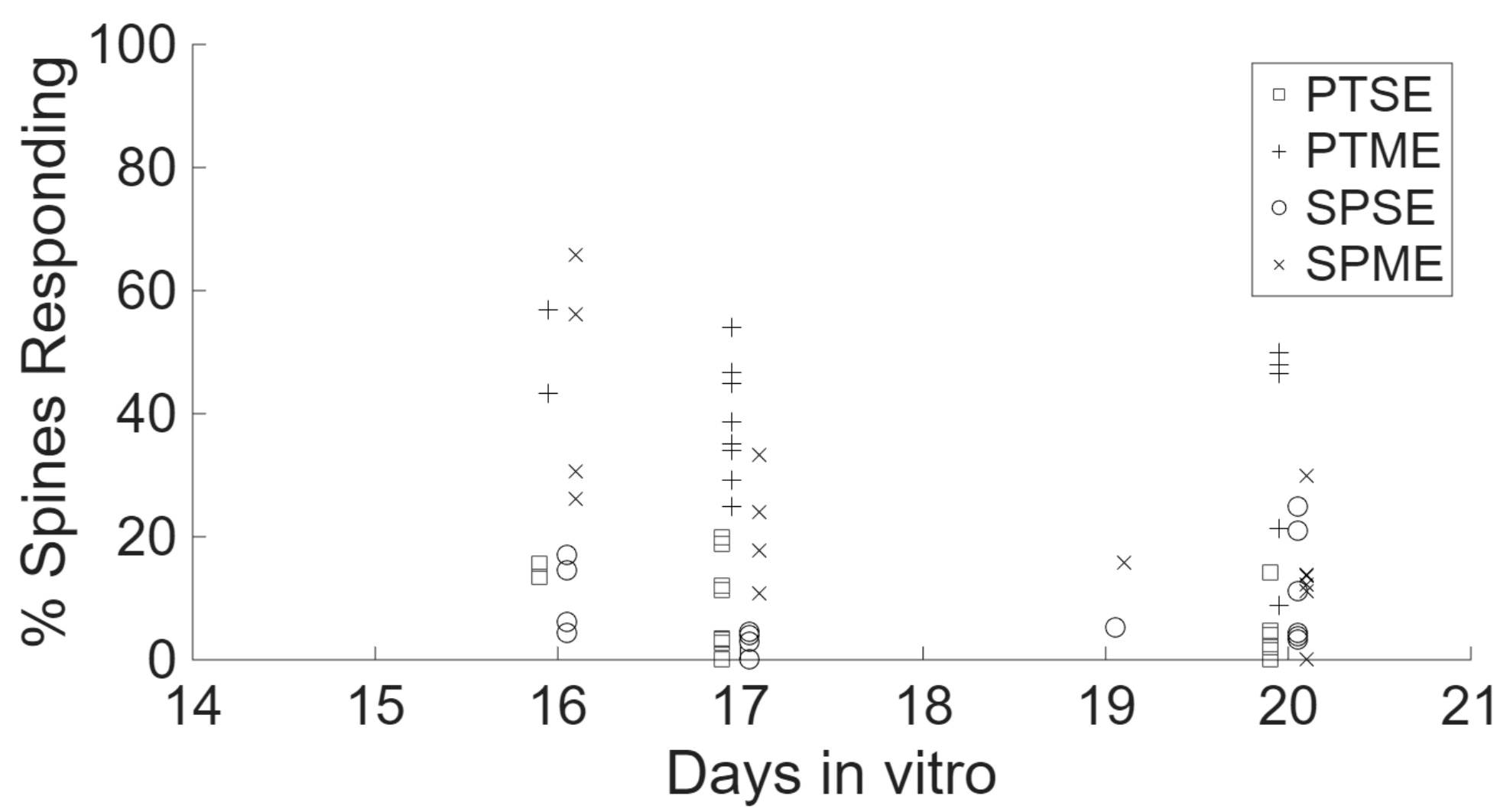

Figure S2: Fraction of spines responding per neuron as a function of maturity for each stimulation protocol. Spearman's ρ = -0.31, -0.19, 0.01, -0.70; p = 0.17, 0.50, 0.97, 0.0036 (PTSE, PTME, SPSE, and SPME protocols, respectively); n = 15 cells for each group.

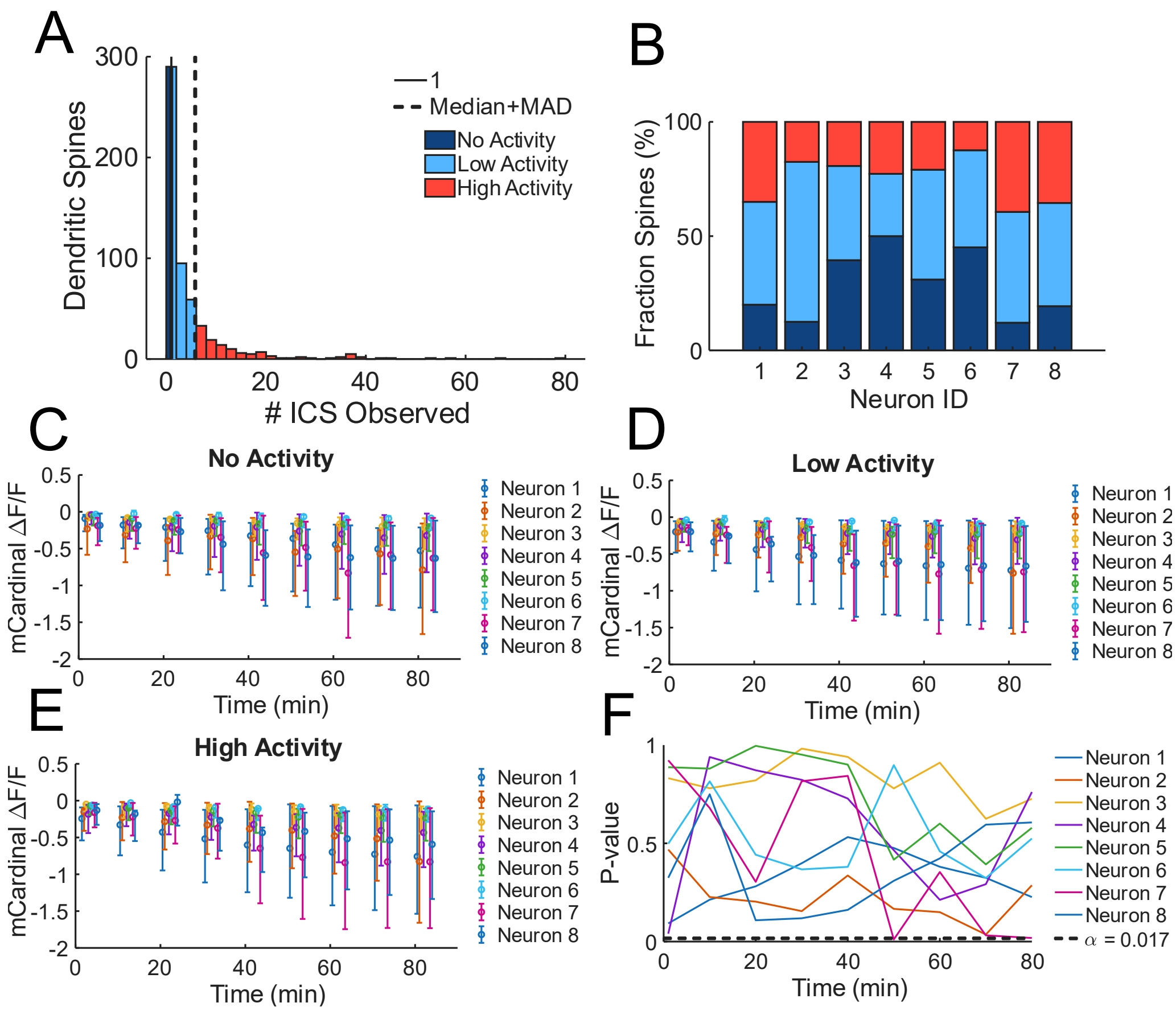

Figure S3: A) Histogram showing the frequency of spiking events across neurons in the experimental group. B) Distribution of spiking activity within individual neurons. C–E) Average change in DS baseline normalized mCardinal fluorescence for each neuron, grouped by activity level, with error bars representing standard deviation. F) P-values from Kruskal–Wallis tests comparing baseline normalized DS mCardinal fluorescence across activity levels over time for each neuron, with the Bonferroni-corrected significance threshold indicated by a dashed line at p = 0.017.

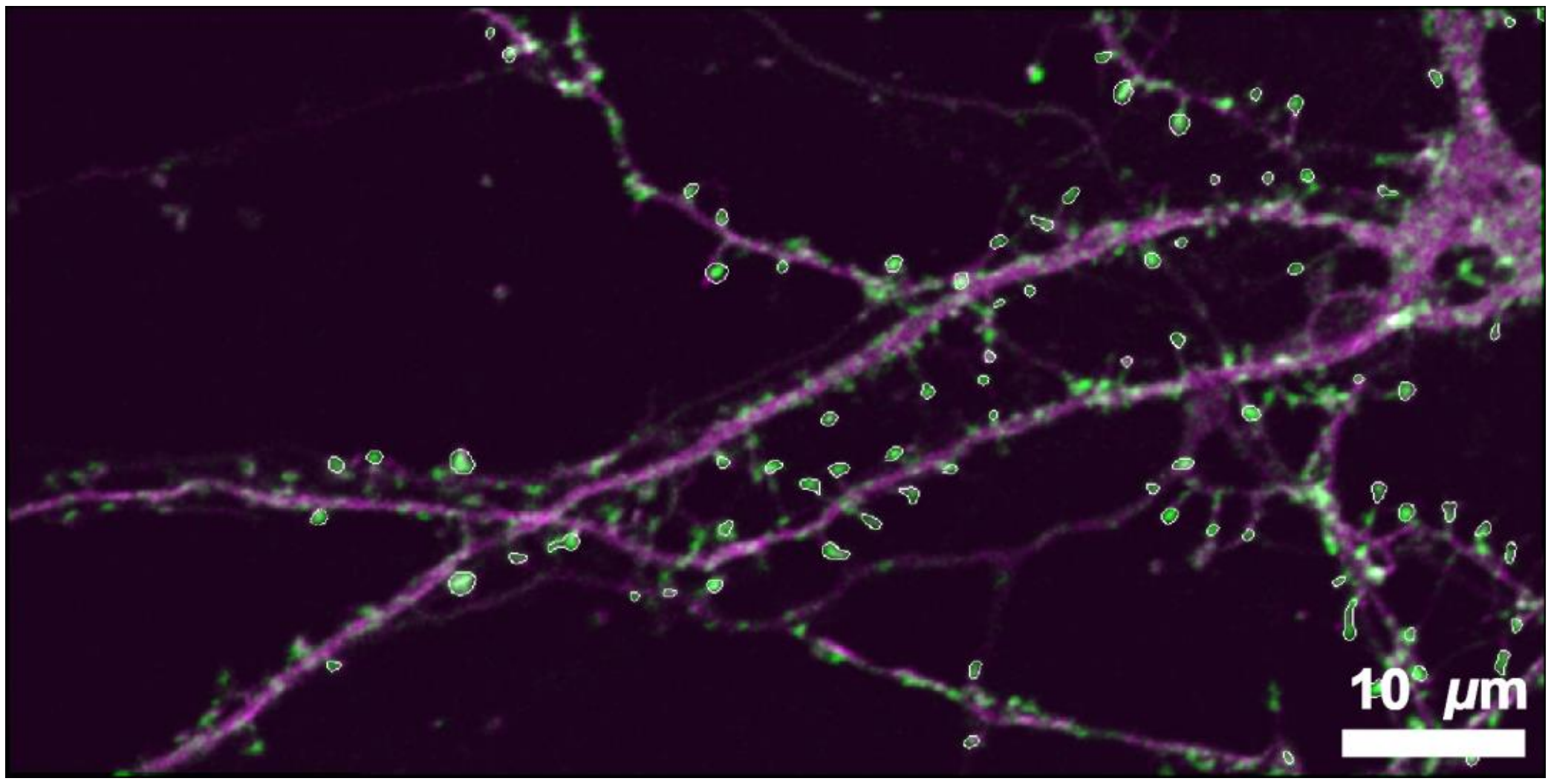

Figure S4: Image of neuron expressing mCardinal-lifeact before (green) and 30 min after (magenta) 20 μM of Latrunculin A. The ROI used for measuring the mCardinal signal are indicated by white outlines. Images were normalized prior to merging the channels, which enhanced the prominence of the brightest regions. The observed increase was not due to an actual rise in actin within the dendrites; rather, it is more intense relative to the spines following application.

Table S1: Pharmacologic inhibitors used to investigate calcium source. The agents Capsazepine, SET2, TRPV3 74a, and RN 1734 were combined as a single inhibitor of TRPV channels, denoted TRP in the manuscript.

Agent	Target(s)	Concentration (μM)
Ruthenium Red (RR)[48-50]	TRPV1-4, CALHM	10
(2R)-amino-5-phosphonovaleric acid (AP5)[21]	NMDAr	50
6-cyano-7-nitroquinoxaline-2,3-dione (CNQX)[21]	AMPAr	20
Gadolinium (Gd^{3+})[59]	Piezo, CALHM	100
Capsazepine[51]	TRPV1	10
SET2[51]	TRPV2	20
TRPV3 74a[51]	TRPV3	10
RN 1734[51]	TRPV4	10

Video S1: Representative video of a neuron responding to the PTSE stimulation protocol. Stimulation is applied 1 minute into the video. The graph below shows the mean intensity of the soma, a representative dendrite, and dendritic spines attached to the parent dendrite.

Video S2: Representative video of a neuron responding to the PTME stimulation protocol. The protocol begins 30 seconds into the video. The graph below shows the mean intensity of the soma, a representative dendrite, and dendritic spines attached to the parent dendrite.
